# Edge Density Imaging Identifies White Matter Biomarkers of Late-Life Obesity and Cognition

**DOI:** 10.14336/AD.2022.1210

**Published:** 2024-08-01

**Authors:** Maxwell Bond Wang, Farzaneh Rahmani, Tammie L. S Benzinger, Cyrus A Raji

**Affiliations:** ^1^Machine Learning Department, Carnegie Mellon University, Pittsburgh, PA, USA.; ^2^Neuroscience Institute, Carnegie Mellon University, Pittsburgh, PA, USA.; ^3^Medical Scientist Training Program, University of Pittsburgh/Carnegie Mellon University, Pittsburgh, PA, USA.; ^4^Mallinckrodt Institute of Radiology, Division of Neuroradiology, Washington University in St. Louis, St. Louis, MO, USA.; ^5^Charles F. and Joanne Knight Alzheimer Disease Research Center (Knight ADRC), Washington University, St. Louis, Missouri, USA.; ^6^Department of Neurology, Washington University in Saint Louis, St. Louis, Missouri, USA

**Keywords:** Edge-Density Imaging, Alzheimer disease Neuroimaging Initiative, Obesity, Structural Connectome

## Abstract

Alzheimer disease (AD) and obesity are related to disruptions in the white matter (WM) connectome. We examined the link between the WM connectome and obesity and AD through edge-density imaging/index (EDI), a tractography-based method that characterizes the anatomical embedding of tractography connections. A total of 60 participants, 30 known to convert from normal cognition or mild-cognitive impairment to AD within a minimum of 24 months of follow up, were selected from the Alzheimer disease Neuroimaging Initiative (ADNI). Diffusion-weighted MR images from the baseline scans were used to extract fractional anisotropy (FA) and EDI maps that were subsequently averaged using deterministic WM tractography based on the Desikan-Killiany atlas. Multiple linear and logistic regression analysis were used to identify the weighted sum of tract-specific FA or EDI indices that maximized correlation to body-mass-index (BMI) or conversion to AD. Participants from the Open Access Series of Imaging Studies (OASIS) were used as an independent validation for the BMI findings. The edge-density rich, periventricular, commissural and projection fibers were among the most important WM tracts linking BMI to FA as well as to EDI. WM fibers that contributed significantly to the regression model related to BMI overlapped with those that predicted conversion; specifically in the frontopontine, corticostriatal, and optic radiation pathways. These results were replicated by testing the tract-specific coefficients found using ADNI in the OASIS-4 dataset. WM mapping with EDI enables identification of an abnormal connectome implicated in both obesity and conversion to AD.

## INTRODUCTION

It is well-known that obesity is not merely an isolated metabolic syndrome, but rather one that interfaces with every organ system in the body. Among these interactions are its proinflammatory effects that are marked with higher levels of inflammatory cytokines, increases blood-brain barrier dysfunction, and neuroinflammation [1-3]. Related to these effects are its links to impaired brain structure and function [4, 5] with earlier studies investigating the association between obesity and gray matter volume, a known imaging biomarker of neurodegeneration in Alzheimer disease (AD) [6-8]. Later work confirmed an independent relationship between obesity and white matter (WM) integrity in adults. Higher body-mass index (BMI), waist circumference or waist-to-hip ratio in mid-life adults is predictive of lower fractional anisotropy (FA) and decreased connectivity in the WM [9-15]. These mechanisms are shown to as least partly mediate the link between obesity and disrupted WM integrity and connectome through reduction of axonal density and demyelination [16-18]. Given that neuroinflammation and WM structural abnormalities are increasingly recognized pathophysiological of AD [19, 20], they might also mediate the relationship between obesity and increased incidence of AD dementia [21-23]. Elucidating obesity’s late-life effects on WM health is of particular interest given conflicting reports of its impact on dementia risk [24-26]. Due to the relative difficulty of treating Alzheimer’s disease compared to launching obesity-related interventions, understanding the precise mechanisms and effects of obesity on neural structure and Alzheimer disease progression is an attractive route of inquiry.

While conventional diffusion-based metrics such as FA quantify changes in the microstructural integrity of specific WM tracts, the use of tractography to assess disruptions in brain structure at a network level remains an underexplored avenue. Implementation of graph-theoretical approaches with diffusion MRI tractography has identified such large-scale relationships, a framework referred to as the structural WM connectome [27]. Using such techniques, decreased WM connectivity with higher BMI has been identified in the brain’s taste-reward circuitry that regulates food intake, as well as in several WM tracts associated with cognition [15, 28-30]. Among the more recently developed graph-theory based analyses, edge density imaging/index (EDI) is a technique that uses tractography to map the anatomical routes that individual pairs of gray matter regions use to communicate with one another [31]. By looking at the average distribution of what routes all pairs take, EDI assesses the location of pathways of connectivity someone’s brain is structurally using [31, 32]. EDI is shown to have a high inter-subject reliability, indicating its ability to reliably detect and distinguish differences between subjects, and is well-conserved regardless of the tractography technique or gray matter parcellation scheme [31-33]. Alterations in the edge density of posterior periventricular WM are better indicative of autism and mild traumatic brain injury in the pediatric population compared to conventional diffusion metrics such as FA, indicating their emerging roles as imaging biomarkers [32, 34, 35].

Given the known association between obesity, WM connectome and risk of AD, we sought to investigate the connectome underpinnings of such relationships using both conventional diffusion metrics and EDI. We used participants in their baseline assessment from the Alzheimer’s Disease Neuroimaging Initiative 2 (ADNI-2) cohort in order to: 1) investigate the relative importance of different WM tracts in the relationship between diffusion metrics or EDI and BMI; 2) investigate the relative importance of different WM tracts in the relationship between diffusion metrics or EDI and conversion to AD; and 3) identify regions with spatial overlap in which higher BMI and risk of AD conversion can both affect the WM connectome.

## METHODS

### Participants

Participants were enrolled from the Alzheimer’s Disease Neuroimaging Initiative (ADNI), [36] the goal of which is to test biomarkers of AD through neuroimaging, genetics, and neuropsychological tests in order to develop new treatments and minimize the lead time of AD clinical trials. ADNI participants are recruited from 58 different sites across the United States and Canada. Due to the time-intensive nature of the tractography-based EDI analyses [32], we selected 60 ADNI participants from screening visits of ADNI-2 (n=50), ADNI-1 (n=6), and ADNI-GO (n=4), based on the following criteria: 1) having a T1-weighted and diffusion-weighted MR scan at their baseline (downloaded through the www.loni.ucla.edu/ADNI/Data/) on September 2019); (2) having normal cognition based on clinical evaluations at the time of baseline scan acquisition; including the Clinical Dementia Rating® scale (CDR®) and the mini-mental state examination (MMSE) scores; and 3) having height and weight measurements available at the time of baseline scan acquisition to compute BMI. Among all participants who met the above criteria we randomly selected 30 representative participants who had stably converted either from normal cognition (n=4) or mild cognitive impairment (MCI) (n=26) to AD (collectively designated as the converter group) as well as 30 age and sex-matched participants who remained cognitively normal in all subsequent available data points as the control group. Participants were excluded if: 1) they had high ischemic score in their diffusion-weighted magnetic resonance imaging, 2) a recent change in medications in the 4 weeks prior to the study, or 3) less than 6 grades of education, or 4) clinical depression.

We used the Open Access Series of Imaging Studies 4 (OASIS-4) cohort which is publicly accessible through the OASIS brain website: (https://central.xnat.org/) as an external validation set for the BMI analyses [37]. Seventeen OASIS-4 subjects were selected from cognitively normal individuals referred with a de novo cognitive complaint and had a structural MRI scan within a maximum of 1 year of a clinical assessment during which a CDR assessment was done [37]. These participants were sex and BMI matched to ADNI participant and were within the same age (age range: 56-85 years, BMI: 26.7±5.8 kg/m2, 9 men and 8 women). As years of education were defined quantitatively in the OASIS-4 versus years of schooling recorded by ADNI, we were unable to replicate results from Model 3.

The study was conducted according to the Good Clinical Practice guidelines and the Declaration of Helsinki. Written informed consent had been obtained from all participants prior to any clinical or imaging protocol. All data acquired as part of this study are publicly available (www.loni.ucla.edu/ADNI/Data/).

### Diffusion and T1-weighted Imaging

All ADNI subjects were scanned according to a standardized protocol as previously described [38]. Briefly, high-resolution T1-weighted structural brain MRI scans were acquired on 3-Tesla MRI scanners through a 3D sagittal volumetric magnetization prepared rapid gradient echo (MP-RAGE) sequence and with the following parameters: repetition time of 2300 ms, flip angle 9°, 24 cm field of view, with a 260 × 240 × 170 acquisition matrix in the x-, y-, and z-dimensions yielding a voxel size of 1×1×1 mm3. Images from different sites were calibrated with phantom-based geometric corrections [39]. Diffusion-weighted MRI (dMRI) images were acquired on a GE scanner using a standardized multi-shell protocol with 5 b0 and 41 diffusion-weighted volumes, a b-value of 1000, and 2.7 × 2.7 × 2.7 mm voxel size as described before [40].

In all OASIS-4 participants, a high-resolution T1 weighted structural brain MRI scans was acquired on a 1.5-Tesla Siemens Vision scanner through a MP-RAGE protocol and dMRI images were acquired with 3 b0 and 18 diffusion-weighted volumes with a b-value of 1000 s/m^2 with a 1.8 x 1.8 x 1.8 mm voxel size.

Diffusion MRI data were preprocessed for eddy current correction and motion artefacts using the FMRIB Software Library (functional magnetic resonance imaging of the brain; FSL, version 5.0.7; Analysis Group, FMRIB, Oxford, UK).

### Diffusion magnetic resonance imaging analyses

We utilized DSI Studio, a publicly available software (http://dsi-studio.labsolver.org/), to generate FA and edge density maps of the selected ADNI participants using a protocol detailed in prior work [32]. We performed motion correction and eddy current distortion correction using the eddy_correct and the FMRIB's Utility for Geometrically Unwarping EPIs (FUGUE) functions from FMRIB Software Library (FSL) v5.0 software [41]. Brain tissue extraction and smoothing were performed using built-in tools from the DSI Studio software package [42]. Cortical regions were parcellated using T1-weighted images and through FreeSurfer version 5.3 and according to the built-in Desikan-Killiany atlas [43, 44]. Parcellated T1-weighted images were then non-linearly registered to the respective dMRI images using DSI Studio [45, 46]. FA maps were generated from generalized Q-sampling (GQI)-reconstructed dMRI images. Deterministic tractography was then performed using the dMRI images to track connections between pairs of gray matter voxels using a GQI motif for fiber tracking [32, 47, 48]. The tracking parameters were as follows: the anisotropy threshold was set to 0.189475. The angular threshold and step size were randomly selected from 15 to 90 degrees and from 0.5 to 1.5 voxels. Tracks with lengths shorter than 10 mm or longer than 300 mm were discarded. A total of two million seeds were placed. In order to obtain average tract connectivity metrics, we parcellated the WM into 45 tracts based on the HCP842 tractography atlas and averaged the FA and EDI indices across each tract to generate a tract-specific statistic.

In neuroanatomy an edge is defined as the anatomic pathway taken between pairs of gray matter regions that are previously established to have reliably detectable connections between them [32]. As a result, the edge density of each white matter voxel (i.e., EDI index) is the number of such connections that passed through each voxel. By counting the number of these connections that pass through each WM voxel, edge density is able to highlight the hub organization of WM connectivity that is unidentifiable through conventional dMRI tractography [49]. Where tractography is unable to route WM connections as a result of edema or aberrant architecture of WM, EDI has shown superior sensitivity to detect alterations in the connectome compared to conventional tensor-based metrics such as FA [34, 35].

We evaluated the quality of brain segmentations, brain tissue extraction, and tract generation through manual inspection and subjects with anatomically implausible results were removed from analyses.

### Statistical Analyses

Baseline variables including tract-specific FA and EDI indices were tested for normality of distribution using the Kolmogrov-Smirnov goodness-of-fit test. If normality of distribution was proven, the independent samples t-test and if not, the Mann-Whitney U test were used for comparisons between overweight and normal weight, obese and non-obese and converter and non-converter groups. P-values were corrected for multiple comparisons using FDR correction through the Benjamini-Hochberg method. For this purpose, the p-values from each model were grouped together and corrected accordingly.

A multiple linear regression was used to find a weighted average of tract-specific statistics (FA and EDI) that was most correlated with BMI [50]. Models to predict BMI were implemented in MATLAB using the Statistics and Machine Learning Toolbox, built first using tract-specific FA/EDI values only (Model 1), and then after adding participant’s age and sex, and years of education (Model 2), as well as participant’s conversion status to AD (Model 3) to the prediction vector. Next, and in order to investigate the optimal combination of FA/EDI metrics associated with the risk of future conversion to AD, a multiple logistic regression with Model 1 incorporating tract-specific FA/EDI indices only and Model 2 adding participant’s age, sex, and years of education to the vector of variables used to predict participant conversion status. Due to the low number of participants converting from normal cognition to AD (n=4) we did not perform separate predictive models in these individuals and those converting from MCI to AD (n=26).

**Table 1:** Description of clinical and cognitive outcomes of the study population.

	Total (n=60)	Normal weight^*^ (n=19)	Overweight or Obese^*^ (n=41)	*p*-value^§^
**Age, years (mean±SD)**	73.8±5.6	78.4±6.3	73.3±5.3	0.350
**Biological Sex**				
**Men (n (%))**	32(53.4%)	12(63.2%)	20(48.8%)	0.299
**Women (n (%))**	28(46.6%)	7(36.8%)	21(51.2%)	
**Education, years (mean±sd)**	15.8±2.9	16.9±3	15.3±2.8	0.051
Conversion to AD^*^^‡^ (Yes/No)				
**Non-converter (n (%))**	30(50%)	10(52.6%)	20(48.8%)	0.781
**MCI to AD (n (%))**	26(43.3%)	7(36.8%)	19(46.3%)	
**Normal to MCI to AD (n (%))**	4(6.7%)	2(10.6%)	2(4.9%)	
**Systolic blood pressure, mmHg (mean±SD)**	134.8±14.5	129.3±12	137.4±15	0.051
**Diastolic blood pressure, mmHg (mean±SD)**	75.5±9.5	74.5±10.1	75.9±9.3	0.589
**Mean arterial pressure, mmHg (mean±SD)**	95.3±9.5	92.8±9.4	96.4±9.5	0.173
**Age, years (mean±SD)**	73.8±5.6	72.5±5.5	75.1±5.5	0.064
**Biological Sex**				
**Men (n(%))**	32(53.4%)	20(67%)	12(40%)	0.038
**Women (n(%))**	28(46.6%)	10(33%)	18(60%)	
**Education, years (mean±SD)**	15.8±2.9	15.7±3	15.8±2.9	0.898
**Systolic blood pressure, mmHg (mean±SD)**	134.8±14.5	136.5±16.5	133.1±12.7	0.370
**Diastolic blood pressure, mmHg (mean±SD)**	75.5±9.5	77.9±10.7	73.1±7.6	0.053
**Mean arterial pressure, mmHg (mean±SD)**	95.3±9.5	97.4±10.7	93.1±7.8	0.081
**BMI, kg/m2 (mean±SD)**	27.7±4.6	27.2±4.8	26.9±4.5	0.830
**BMI categories^‡^, U, N, Ow, Ob, Mo (n)**	0/19/31/9/1	0/10/14/6/0	0/9/17/3/1	0.504
**Overweight**				
**Overweight (n (%))**	41(68.3%)	20(67%)	21(70%)	0.788
**Non-overweight (n (%))**	19(31.7%)	10(33%)	9(30%)	
**Obese**				
**Obese (n (%))**	10(16.7%)	6(20%)	4(13.3%)	0.481
**Non-obese (n (%))**	50(83.3%)	24(80%)	26(86.7%)	

* Being overweight or obese was defined based on a BMI of 25 or above; Conversion to AD: Participants who converted from normal cognition or mild cognitive impairment to Alzheimer disease (AD).

§ Bold values indicate significant tests.

‡ U: underweight; N: normal; Ow: overweight; Ob:obese; Mo: Morbidly obese; AD: Alzheimer disease; BMI: Body mass index; ADNI subjects: 4081,2007,2047,2106,4097,4119,4162,4245,4240,4254,4234,4421,4441,4458,4488,4507,4459,4516,4385,4279,4503,4506, 4555,4387,4402,4584,4620,4637,4585,4644,4350,4712,4729,4757,4765,4688,4290,4659,4872,4888,4900,4902,4928,4943,4936,4952,4951, 4558,4276,4335,5031,0123,2398,4157,0934,4844,0778,1346,0671,0610

In order to externally validate the weights identified to connect FA and EDI to BMI, we used 17 cognitively normal participants from the OASIS-4 dataset with the same sex, age and BMI distribution as ADNI participant (p-values of between group comparisons >0.05). Next, we took the optimized weights for each tract, based on models run on the ADNI database and calculated the weighted average of FA or EDI from the OASIS-4 participants and ran a Pearson’s correlation model between the resulting metric and BMI of OASIS participants. Separate models were run for FA and EDI metrics, once without and once with OASIS-4 participants age and sex as covariates. A significant correlation between the weighted sum or FA or EDI metric and BMI of OASIS-4 participants would validate results from the larger ADNI sample.

Statistical significance of regression analyses were tested using Bartlett’s chi-squared test with Lawley’s modification to compensate for multiple comparisons correction [51]. The Bartlett’s test allows for an unbiased comparison between the variance of the test variable (here BMI or conversion status to AD) and the regression metric. Moreover, the Bartlett’s χ^2^ can be used to compare regression performance between two models. A p-value below 0.05 was considered statistically significant in all tests.

**Figure 1.**
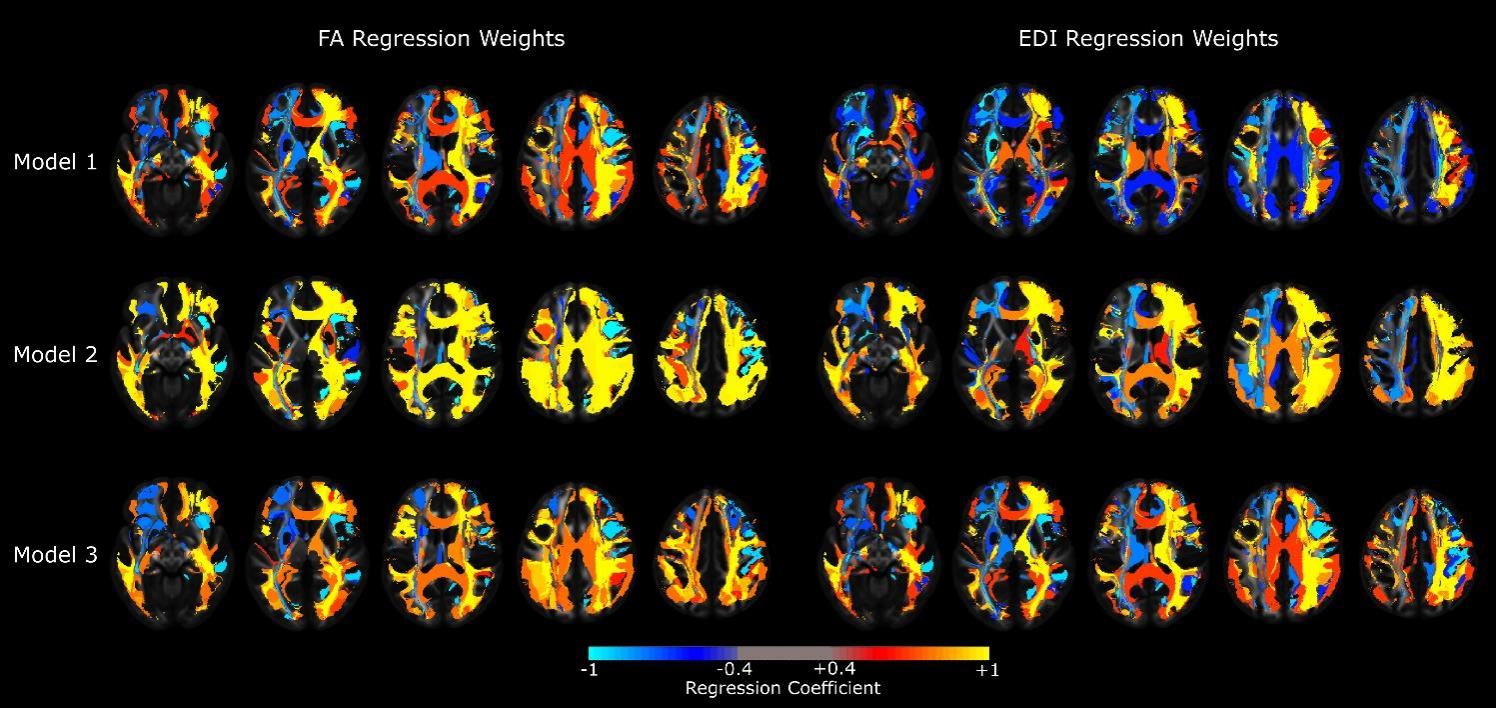
**Color-coded map of weights from regression models predicting BMI using tract-specific FA or EDI values**. *Footnote:* Colors reflect the sign and magnitude of the coefficient of each WM tract in each model. Model 1 included the FA/EDI values only, while Model 2 incorporated age, sex and years of education, and Model 3 additionally included conversion status to AD in the prediction vector. *Abbreviations*: FA: fractional anisotropy; EDI: edge-density imaging index; BMI: body-mass index

## RESULTS

**Table 2:** White matter tracts with largest contributions to the relationship between fractional anisotropy (FA) and body mass index (BMI).

Model 1		Model 2		Model 3	
**Tracts**	Regression Coefficients*	Tracts	Regression Coefficients*	Tracts	Regression Coefficients*
**Corticothalamic pathway (right)**	-1.2	Corpus Callosum	-1.2	Frontal aslant tract (right)	1.01
**Temporopontine tract (left)**	1.10	Anterior commissure	1.09	Cortical U_Fibers (right)	0.89
**Cortical U_Fibers (right)**	0.90	Vertical occipital fasciculus (left)	-1.00	Arcuate fasciculus (left)	0.83
**Frontopontine tract (right)**	0.79	Temporopontine tract (left)	0.99	Corticothalamic pathway (right)	0.77
**Arcuate fasciculus (left)**	-0.78	Extreme capsule (left)	-0.91	Optic radiation (left)	0.76
**Cingulum (right)**	0.75	Cortical U_Fibers (left)	0.79	Inferior frontoccipital fasciculus (left)	0.75
**Corticothalamic pathway (left)**	0.73	Frontopontine tract (left)	-0.76	Corpus Callosum	0.69
**Inferior frontooccipital fasciculus (left)**	0.72	Corticostriatal pathway (right)	0.74	Corticostriatal pathway (left)	0.68
**Frontal aslant tract (right)**	0.70	Corticospinal tract (left)	0.72	Frontopontine tract (right)	0.61
**Fornix (left)**	0.69	Middle longitudinal fasciculus (right)	0.69	Fornix (left)	0.58
**Optic radiation (left)**	-0.68	Acoustic radiation (right)	0.59	Acoustic radiation (right)	0.56
**-**	-	Extreme capsule (right)	0.58	-	-
*P-value* **	0.072	*P-value* **	0.055	*P-value* **	0.063
**r‡**	0.9076	r‡	0.9284	r‡	0.9319
**Bartlet’s χ2§**	59.5	Bartlet’s χ2§	64.6	Bartlet’s χ2§	64.95

* Relationship between FA and BMI with no covariates (Model 1), age, sex and education as covariates (Model 2), and age, sex, education and AD conversion status as covariates (Model 3). White matter tracts in each model were rank-ordered based on absolute values of standardized regression coefficients/weights and only tracts with absolute coefficients above the 3^rd^ quartile are demonstrated.

** Bartlett’s test p-value after Lawley’s modification

‡ Pearson’s correlation coefficient

§ Bartlett’s chi-squared test value with Lawley’s modification

Compared to normal weight individuals, overweight or obese participants were not different in tract-specific FA or EDI indices in any of the studied WM tracts (*p-value>0.05*). Converters had an average lower FA in the left frontopontine tract (*p-value:0.045*, Cohen’s D: 0.302), left optic radiation (*p-value:0.033*, Cohen’s D: 0.880), left parietopontine tract (*p-value:0.033*, Cohen’s D: 0.662), and bilateral inferior frontoccipital fasciculi (IFOF) (*p-value:0.0225*, Cohen’s D: 0.834 and 0.031 for the left and right tracts respectively). Converters also demonstrated lower EDI in the left optic radiation (*p-value:0.045*, Cohen’s D= 0.880), compared to non-converter individuals. There were no statistically significant differences in FA or EDI of any of the studied tracts between participants who converted from normal cognition to AD, and those who converted from MCI to AD (between group post-hoc *p-values<0.05*).

**Figure 2.**
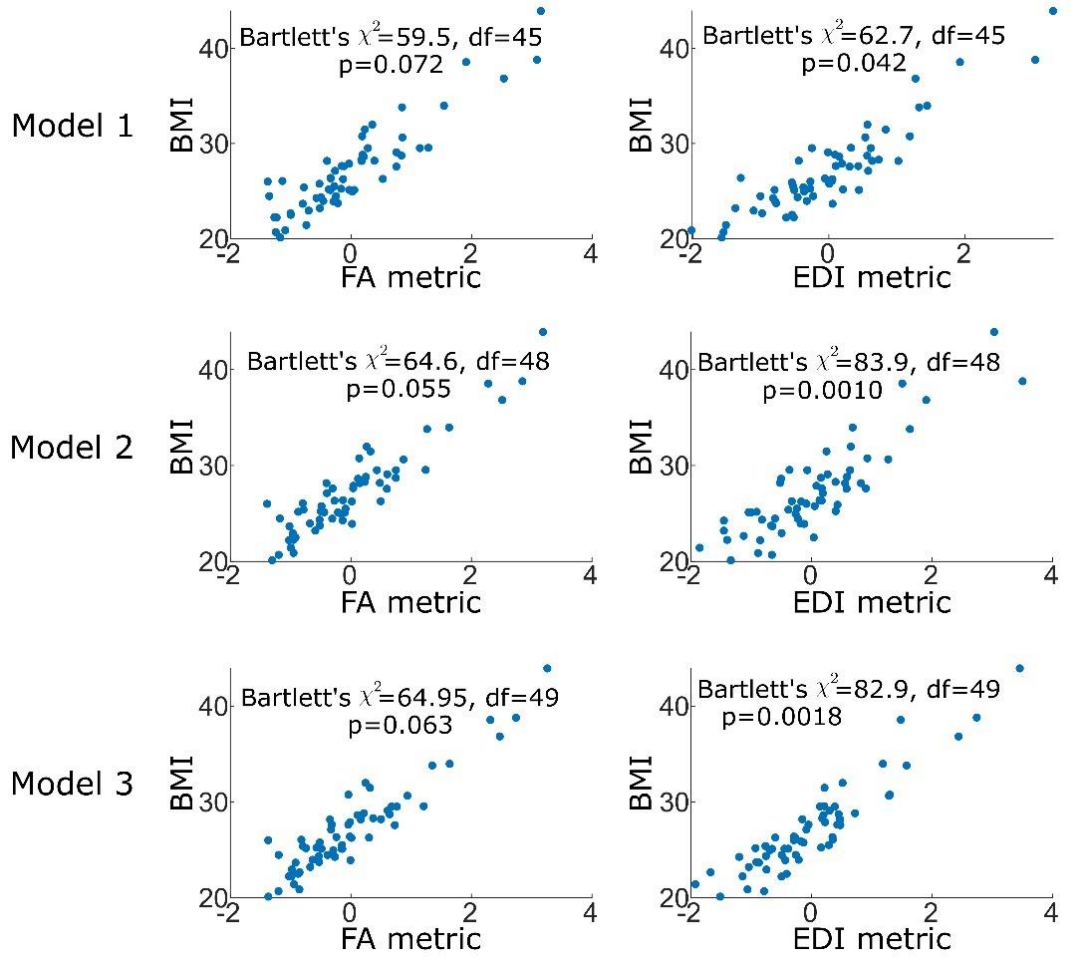
**Performance of the regression model is demonstrated as the relationship between the regression metric and BMI**. Footnote: Statistics in each model are demonstrated as the Bartlett’s chi-square and p-value. The performance of the EDI to BMI model in an external dataset is shown in the bottom-left and evaluated using Pearson’s correlation. Abbreviations: FA: fractional anisotropy; EDI: edge-density imaging index; BMI: body-mass index; df; degree of freedom, χ2: Chi-square

**Figure 3.**
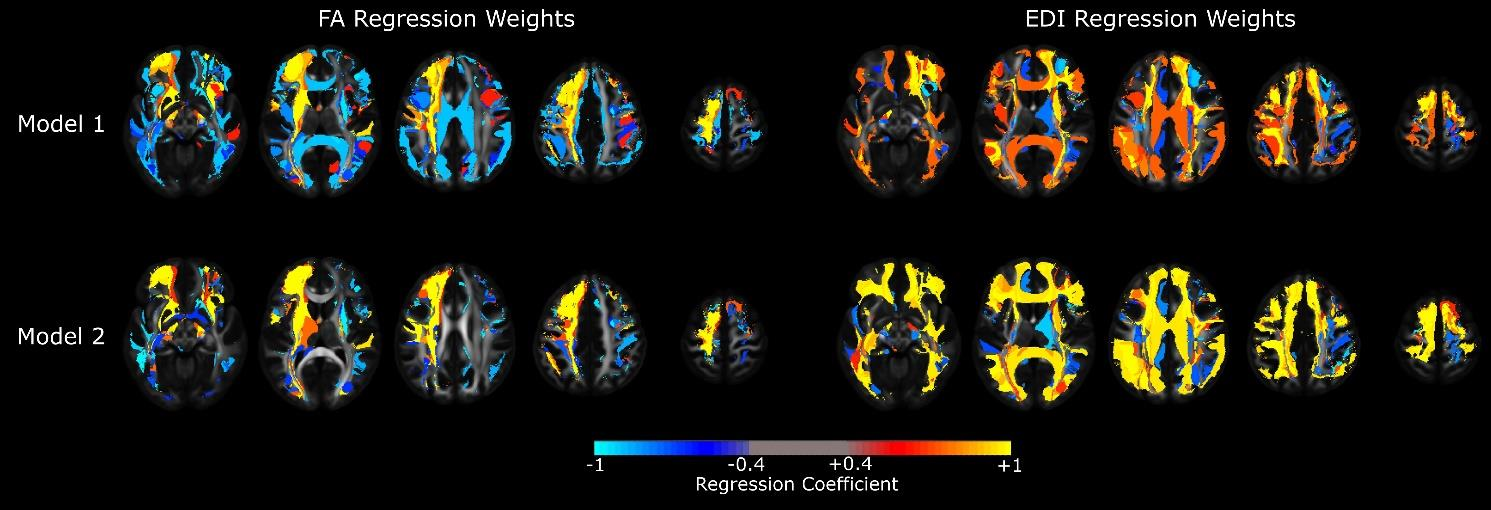
**Color-coded map of coefficient weights from regression models predicting conversion to AD using tract-specific FA or EDI values**. Footnote: Colors reflect the sign and magnitude of the coefficient of each WM tract in each model. Model 1 included the FA/EDI index values only, while Model 2 incorporated age, sex and years of education in the prediction vector. Abbreviations: FA: fractional anisotropy; EDI: edge-density imaging index; AD: Alzheimer disease

### Regression analyses

The average FA and EDI values for each of the 45 studied tracts were used to compute a regression metric comprised of a weighted average of values with maximum correlation to individual BMI values. For each connectivity metric (FA or EDI) the regression Model 1 was conducted using a linear combination of tract-specific values and BMI, while Models 2 and 3 included addition of age, sex, years of education (Model 2), as well as conversion status to AD (Model 3) to the linear prediction vector. Figure 1 demonstrates the regression weights of WM tract from each model and Figure 2 demonstrates the relationship between the weighted FA or EDI metric from each model and BMI.

**Table 3:** White matter tracts with largest regression contributions to the relationship between edge density imaging (EDI) metrics and body mass index (BMI).

Model 1		Model 2		Model 3	
Tracts	Standardized Regression Coefficients*	Tracts	Standardized Regression Coefficients*	Tracts	Standardized Regression Coefficients*
Cingulum (left)	1.02	Frontopontine tract (left)	2.13	Frontopontine tract (left)	2.11
Frontopontine tract (left)	-1.01	Arcuate fasciculus (left)	1.61	Arcuate fasciculus (left)	1.57
Anterior commissure	0.99	Cingulum (left)	-1.38	Anterior commissure	-1.37
Vertical occipital fasciculus (right)	-0.96	Anterior commissure	-1.37	Cingulum (left)	-1.37
Corticostriatal pathway (left)	0.95	Superior longitudinal fasciculus (right)	-1.32	Superior longitudinal fasciculus (right)	-1.35
Extreme capsule (left)	0.95	Posterior commissure	-1.20	Posterior commissure	-1.17
Fornix (right)	0.94	Fornix (right)	-1.10	Fornix (right)	-1.06
Arcuate fasciculus (right)	-0.91	Corticostriatal pathway (right)	-1.06	Corticostriatal pathway (right)	-1.05
Posterior commissure	0.82	Inferior longitudinal fasciculus (left)	0.88	Inferior longitudinal fasciculus (left)	0.88
Frontopontine tract (right)	-0.79	Extreme capsule (right)	-0.87	Extreme capsule (right)	-0.86
Acoustic radiation (right)	0.77	Optic radiation (left)	0.85	Optic radiation (left)	0.85
Temporopontine tract (right)	0.73	Superior longitudinal fasciculus (left)	0.75	Vertical occipital fasciculus (right)	0.75
*P-value* **	0.042	*P-value* **	<0.0001	*P-value* **	0.0018
**r‡**	0.9079	**r‡**	0.9574	**r‡**	0.9581
**Bartlet’s χ2§**	62.7	**Bartlet’s χ2§**	83.9	**Bartlet’s χ2§**	82.9

* Relationship between EDI and BMI with no covariates (Model 1), age, sex and education as covariates (Model 2), and age, sex, education and AD conversion status as covariates (Model 3). White matter tracts in each model were rank-ordered based on absolute values of standardized regression coefficients/weights and only tracts with absolute coefficients above the 3^rd^ quartile are demonstrated.

** Bartlett’s test p-value after Lawley’s modification.

‡ Pearson’s correlation coefficient.

§ Bartlett’s chi-squared test value with Lawley’s modification EDI: edge density imaging; BMI: body-mass index

**Figure 4.**
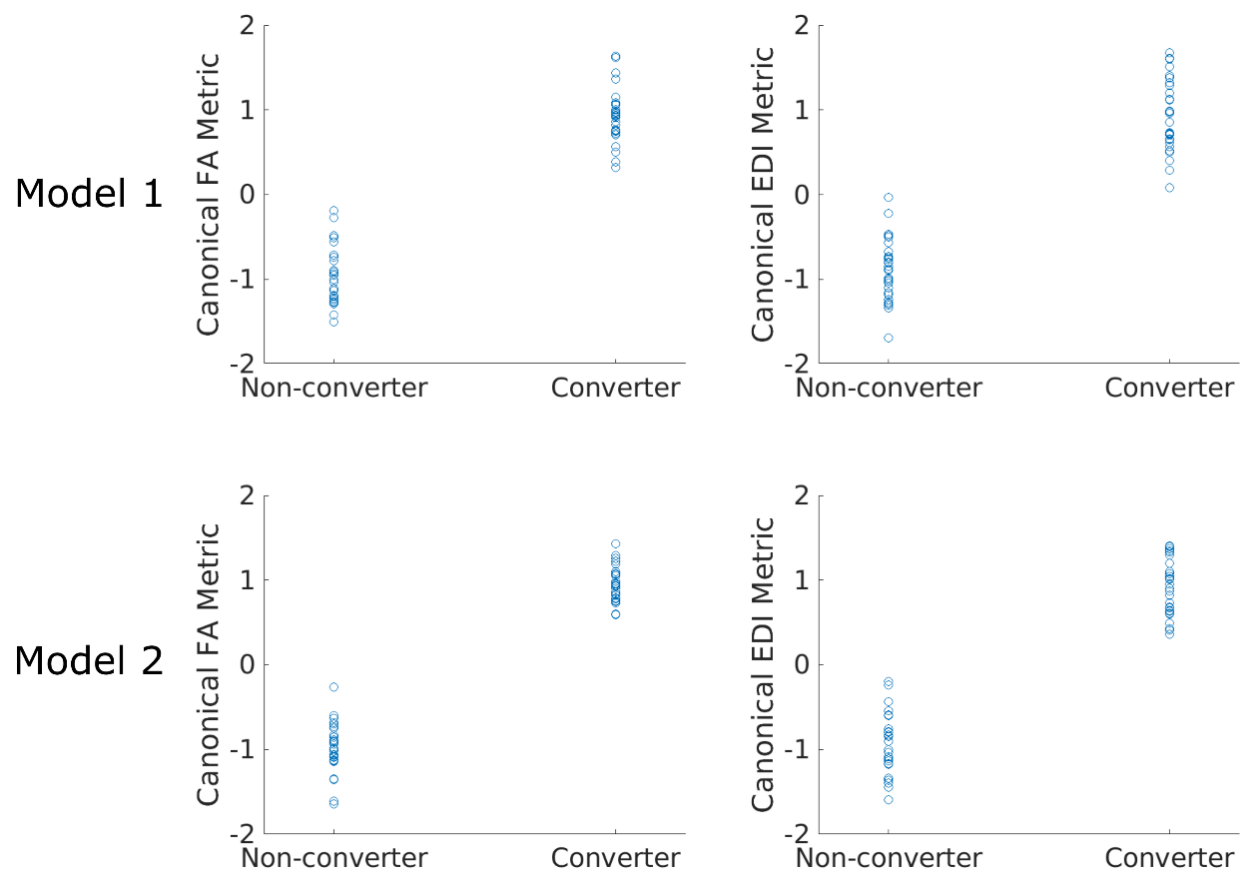
**Performance of the converter model is demonstrated as the relationship between the regression metric and conversion status**. Footnote: Statistics in each model are demonstrated as the Bartlett’s chi-square and p-value. Abbreviations: FA: fractional anisotropy; EDI: edge-density imaging index; df; degree of freedom, χ^2^: Chi-square

FA in the right frontal aslant tract, right corticothalamic pathway, right frontopontine tract, right acoustic radiation, the right cortical U-fibers, and the left arcuate fasciculus (AF), left IFOF, left corticostriatal pathway, left temporopontine tract, left fornix, the left optic radiation, as well as the corpus callosum were the WM tracts with highest absolute weights in the regression model that was best correlated with BMI across all three models (Table 2). Similarly, EDI in the bilateral AF, bilateral frontopontine, bilateral corticostriatal, bilateral superior longitudinal fasciculi (SLF), the anterior commissure, as well as the cingulum, frontopontine tract, inferior longitudinal fasciculus (ILF), and optic radiation on the left, and the temporopontine tract, vertical occipital fasciculus (VOF) and the fornix on the right, comprised the most important set of variates to predict BMI across all three models (Table 3). No statistically significant result was observed in models using apparent diffusion coefficient, radial diffusivity, or axial diffusivity in predicting either the BMI or conversion. We did not find major correlations between age and sex to our imaging metrics, FA, or EDI.

Supplementary Figure 1 demonstrates the results from correlation models applying coefficients from models using ADNI (Fig. 1 and 2) to tract-specific FA and EDI values from the OASIS-4 sample.

### Converter status analysis

We used the average FA and EDI of the studied 45 WM tracts to find the optimal linear combination of tracts that differentiated the converter and non-converter groups. Like the regression analyses, Model 1 was conducted using the connectivity metrics (FA and EDI) only, while Model 2 incorporated the participant’s age, sex and education in the prediction vector. Coefficients of each WM tract contributing to the regression metric are demonstrated in Figure 3, while Figure 4 demonstrates how FA and EDI metrics differ between converter and non-converter groups. Tracts in which FA had the highest weight in the regression metric that best predicted the conversion status included the optic radiation, IFOF, uncinate fasciculus (UF), middle longitudinal fasciculus (MLF), and the corticospinal tract on the right, as well as the corticostriatal tract, frontopontine tract, the SLF and the fornix on the left (Table 4). Similarly, the EDI in the left frontopontine, left frontal aslant tract and the left VOF, as well as the right corticostriatal tract, right AF, and the right optic radiation were among most important elements in the regression model best differentiating converters from non-converters (Table 4).

**Table 4:** White matter tracts with largest regression contributions to the relationship between FA and EDI with BMI.

FA			EDI				
Tracts	Standardized Regression Coefficients*	Tracts	Standardized Regression Coefficients*	Tracts	Standardized Regression Coefficients*	Tracts	Standardized Regression Coefficients*
Corticostriatal pathway (left)	1.39	Corticostriatal pathway (left)	2.13	Frontopontine tract (left)	1.05	Frontopontine tract (left)	1.11
Optic radiation (right)	1.19	Inferior frontoccipital fasciculus (right)	1.61	Corticostriatal pathway (right)	0.91	Corpus callosum	0.94
Inferior frontoccipital fasciculus (right)	1.15	Uncinate fasciculus (right)	-1.38	Corpus callosum	0.58	Corticostriatal pathway (right)	0.85
Uncinate fasciculus (right)	0.94	Optic radiation (right)	-1.37	U_Fiber (left)	0.48	Frontal aslant tract (left)	0.73
Middle longitudinal fasciculus (right)	0.92	Corticospinal tract (right)	-1.32	Frontal Aslant tract (left)	0.46	Posterior commissure	0.58
Frontopontine tract (left)	0.84	Frontopontine tract (left)	-1.20	Middle longitudinal fasciculus (right)	0.37	Arcuate fasciculus (right)	0.57
Corticospinal tract (right)	0.75	Superior longitudinal fasciculus (left)	-1.10	Arcuate fasciculus (right)	0.36	Temporopontine tract (left)	0.54
Superior longitudinal fasciculus (left)	0.70	Middle longitudinal fasciculus (right)	-1.06	Optic radiation (left)	0.31	Superior longitudinal fasciculus (right)	0.45
Fornix (left)	0.54	Vertical occipital fasciculus (right)	0.88	Optic radiation (right)	0.30	Optic radiation (right)	0.44
Frontopontine tract (right)	0.45	Fornix (left)	-0.87	Inferior frontoccipital fasciculus (right)	0.23	Frontopontine tract (right)	0.43
Parietopontine tract (left)	0.41	Corticothalamic pathway (left)	0.85	Vertical occipital fasciculus (left)	0.20	Vertical occipital fasciculus (left)	0.35
-	-	Frontopontine tract (right)	0.75	-	-	-	-
P-value**	0.0014	P-value**	<0.001	P-value**	0.019	P-value**	0.0084
Bartlet’s χ2§	78.7	Bartlet’s χ2§	92.4	Bartlet’s χ2§	66.8	Bartlet’s χ2§	74.5

* Relationship between FA and BMI with no covariates (Model 1), age, sex and education as covariates (Model 2), and age, sex, education and AD conversion status as covariates (Model 3). White matter tracts in each model were rank-ordered based on absolute values of standardized regression coefficients/weights and only tracts with absolute coefficients above the 3^rd^ quartile are demonstrated.

** Bartlett’s test p-value after Lawley’s modification

§ Bartlett’s chi-squared test value with Lawley’s modification

## DISCUSSION

We used cognitively normal ADNI participants at baseline to identify signature WM tracts in which the EDI and FA indices were related to BMI and AD conversion. Results from BMI models were further validated using 17 cognitively normal OASIS-4 participants. We identified the most important WM tracts implicated in such associations by comparing the regression weights across tracts. Our findings revealed: 1) major crossing-fiber rich, periventricular, commissural and projection WM fibers to be among the most important tracts in linking BMI and WM connectivity (FA and EDI), 2) WM areas in which EDI index was more important than FA in predicting BMI, that were typically located in the non-periventricular regions such as the bilateral frontal poles and the centrum semi-ovale, 3) overlap between the WM correlates of BMI and conversion to AD; that were most prominent in the corticostriatal pathway, frontopontine tract, optic radiation, AF and VOF for EDI-based models, and in the corticostriatal pathway, frontopontine tract, optic radiation, IFOF and fornix for FA-based models.

### EDI Maximizes Precision in Identifying Signature WM

Tracts Associated With BMI and Conversion to AD

Our study represents an initial investigation using the EDI index to reveal WM correlates of BMI and conversion to AD. Conventional DTI techniques such as tractography can provide insights into the average WM integrity (FA, MD, etc.), Additional quantitative insights can be obtained using WM tract density, or WM connectivity in specific WM regions [52]. While these approaches successfully reveal changes in WM from a microstructural standpoint, they do not reflect how alterations in microstructure translate to the overall organization of the WM connectome. One of most successful methodologies to investigate changes in the WM connectome are graph-theory based approaches where different cortical regions serves as nodes and the WM fibers serve as edges of a given connectome matrix [27]. EDI complements the mentioned approach by mapping the architecture of these edges within known WM fibers, thereby providing information of the relative importance of each fiber in the WM connectome. We refined this approach through regression analyses that allowed us to create a linear weighted biomarker of fiber tractography that linked EDI to BMI. This approach is advantageous compared to typical correlation methods such as Pearson’s. While both methods investigate the relationship between two variables, Pearson correlation can only compare two variables irrespective of any cross-covariance between variables [53]. Additionally, conduction of ordinary correlation between an exhaustive set of independent variables and one dependent variable, such that happens with multiple WM tracts, creates the issue of multiple comparisons and mandates reductive approaches to interpret the data [54]. By creating a multidimensional variable fiber-specific FA and EDI values, these regression analyses can explore such relationships without the limitations of typical correlation.

### WM Tracts Related to BMI and Conversion Inform Shared Pathophysiology between Obesity and AD

Prior work has demonstrated an edge-density rich nexus in the posterior WM region that is spatially aligned with the corpus callosum, internal capsules, and the periventricular WM area [31]. These areas encompass all three types of commissural (interhemispheric), associational (intrahemispheric), and projectional (corticofugal) streamlines and correspond to the periarterial WM which is known to be enriched with crossing fibers [55]. Similarly, we found the most important WM trajectories in the regression model with EDI and BMI to be located at these edge-density rich areas; including the anterior and posterior commissure (commissural fibers), the frontopontine and corticostriatal tracts (projectional fibers), as well as the fornix, cingulum, AF, SLF and ILF (associational fibers).

WM fibers that contributed significantly to the regression model predicting BMI and conversion to AD overlapped in the frontpontine, corticostriatal, optic radiation, AF and the VOF pathways in models using the EDI index and in the frontpontine, corticostriatal, optic radiation, IFOF and fornix pathways in models using FA. Disrupted structural connectome in these fibers, notably in the fornix, AF, and IFOF have been noted with conversion to AD from mild cognitive impairment [56-58, 58]. Similarly we have shown changes in the connectivity in these WM fibers with increasing BMI in healthy older adults [15]. These associational WM fibers connect the medial temporal lobe to other parts of the limbic system. Dysconnectivity in these fibers, such that reflected by reduced edge density, might lead to circuit hyperexcitability, acceleration of local age-related tau deposition and eventually furthering amyloid beta deposition in the originating cortical structures such as the posteromedial cortex [59]. Together with current study, our findings suggest that the reduced edge-density associated with increasing BMI in adults may perpetuate AD pathology through hyperexcitability [60]. This hypothesis needs to be further investigated in future work.

The association between optic radiation connectome, BMI and AD conversion is a novel finding and might be explained by considering the reduced density of the ganglion cell layer of the retina in patients with preclinical AD, resulting in retrograde optic radiation disconnection and visual cortical damage [61, 62]. Recent evidence also points to a link between reduced retinal nerve fiber layer thickness and reduced cognitive abilities in overweight and obese adults [63, 64]. Furthermore, obesity is strongly predictive of a variety of retinal pathologies such as diabetic retinopathy and optic neuropathy, furthering support of the relationship between BMI and connectivity in the optic radiation. Our results connecting BMI to AD conversion through the optic radiation and the VOF stimulate future research to elucidate different aspects of this relationship.

The corticostriatal and frontopontine pathways are among the most fiber-dense corticofugal WM structures in which our study demonstrated association between reduced EDI and FA with increasing BMI [65, 66]. The corticostriatal pathway is part of the cortico-striato-pallido-thalamic-cortical re-entrant circuit that is highly asymmetric and implicated in a variety of striatum-related neurodegenerative conditions [67]. This pathway is also implicated in the taste-reward circuitry connecting the higher-order foci of control in the orbitofrontal and anterior cingulate cortex to the striatum and from there to the hypothalamus, thereby controlling the feeding behavior and response to food-related cues [68]. This implicates a possible link between abnormal corticostriatal connectome, such that detected through reduced EDI index, uninhibited signal transduction to the lateral hypothalamus, which is the key hypothalamic structure implicated in food choice, and craving and eating behavior [68].

Together with evidence from literature our findings support the presence of shared pathophysiology between obesity and AD. Given ample empirical and scientific evidence on the association between obesity and risk for AD, our findings provide new insights on WM underpinnings of this relationship. On one hand prevention or treatment of obesity could mitigate dysconnectivity-related changes in the medial temporal lobe and reduce the risk of late-life dementia, and on the other hand weight loss could prevent connectome changes in the WM of the taste-reward circuitry preventing unhealthy food choices and excessive craving.

### Strengths and limitations

The use of a well-defined cohort such as the ADNI-2 and ADNI-GO, allowed us to empirically identify WM connectome correlates of BMI and conversion. Furthermore, findings from the models using BMI were externally validated using cognitively normal OASIS-4 participants. However, these findings might not be applicable to a general patient population as these participants were drawn from referral clinics have relatively fewer comorbidities such as advanced ischemic heard and cerebral vascular disease. The innovation of high-performance computing based methods such as the Massively Parallel, Portable, and Reproducible Tractography (MaPPeRTrac) (https://github.com/LLNL/MaPPeRTrac) pipeline allows for high throughput EDI processing, enabling us to verify findings from the current study in larger populations [69].

Another limitation is the use of single shell diffusion which is known to be less reproducible than multi shell studies [37]. More broadly, tractography is known to have varied sensitivity/specificity across neural anatomy: while this study focuses on analyzing variance within each tract and connecting it to biomarkers of interest, this point still stands when comparing data from one anatomical region to another. We hope these results can inspire investigations using more robust imaging methods.

### Conclusions

We applied a novel imaging biomarker of WM connectome, edge density imaging, to study the relative importance of WM structures in the association between WM connectome, BMI, and conversion to AD. We identified several edge-density rich WM fibers, left more than right, both in the periventricular and non-periventricular areas that were implicated in this association and validated these tracts’ relationship to BMI in an external dataset. These fibers are implicated in the reward network of the brain and hedonic food intake implicating a positive feedback loop between obesity and WM disconnection that perpetuates further WM damage and abnormal food intake.

### Data availability

Data used in this study is publicly accessible through Alzheimer Disease Neuroimaging Initiative (ADNI) (www.loni.ucla.edu/ADNI/Data/).

## Supplementary Materials

The Supplementary data can be found online at: www.aginganddisease.org/EN/10.14336/AD.2022.1210.


