# Supplementary material for: Edge Density Imaging Identifies White Matter Biomarkers of Late-Life Obesity and Cognition
Source: Aging Dis. 2024 Aug 1;15(4):1899–912. doi: 10.14336/AD.2022.1210 (PMC11272213; doi:10.14336/AD.2022.1210)
Supplement: Supplementary file 1 [file AD-15-4-1899-s.pdf]

SUPPLEMENTARY DATA

Edge Density Imaging Identifies White Matter Biomarkers of Late-Life Obesity and Cognition

Maxwell Bond Wang, Farzaneh Rahmani, Tammie L. S. Benzinger, Cyrus A. Raji

© 2022. Wang MB et al. Published online at http://www.aginganddisease.org/EN/10.14336/AD.2022.1210

SUPPLEMENTARY DATA

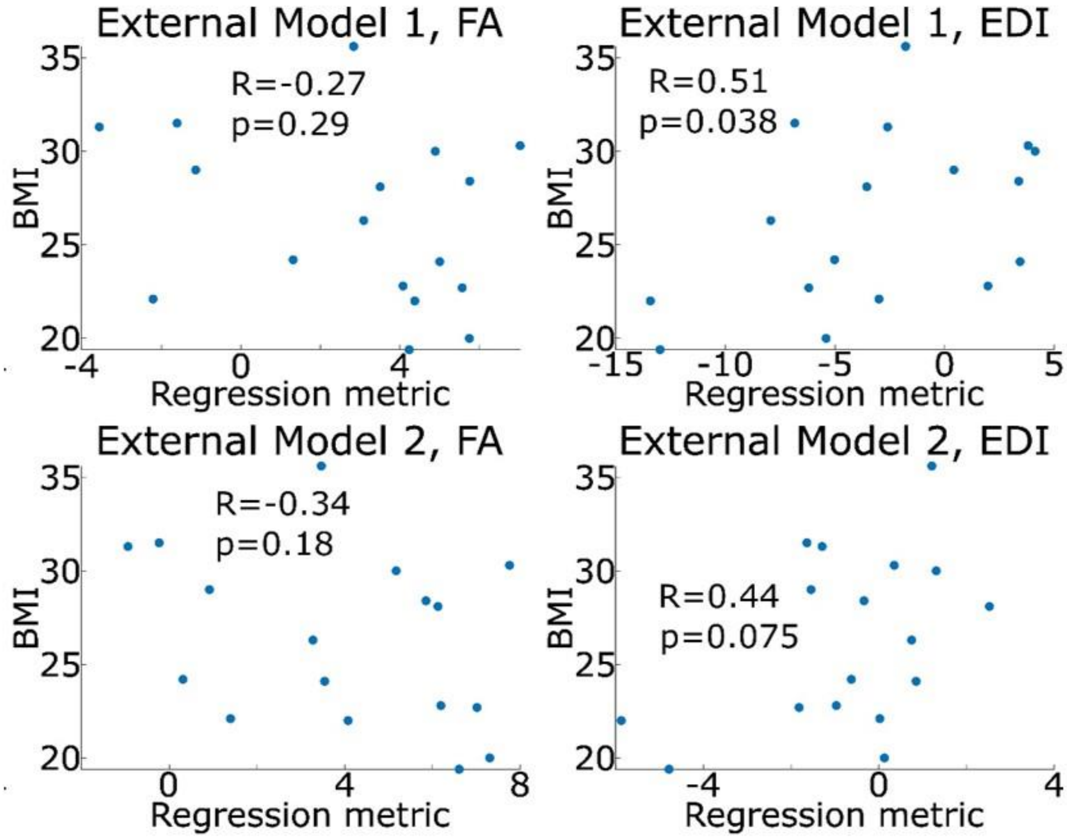

Supplementary Figure 1. Correlation results from validation of coefficients from ADNI-based models using OASIS-4 participants.

Footnote: Correlation statistics are represented by R and p-vales for each model. Colors reflect the sign and magnitude of the coefficient of each WM tract in each model. Model 1 included the weighted sum of FA/EDI values only, while Model 2 incorporated age, sex as covariate

Abbreviations: FA: fractional anisotropy; EDI: edge-density imaging index; BMI: body-mass index

© 2022. Wang MB et al. Published online at http://www.aginganddisease.org/EN/10.14336/AD.2022.1210
